# Advancing organ-on-chip systems: the role of microfluidics in neuro-cardiac research

**DOI:** 10.1016/j.crphar.2025.100227

**Published:** 2025-07-03

**Authors:** Maria João Ferreira, Sarah Colombani, Albin Bernardin, Alain Lacampagne, Jean-Luc Pasquié, Pedro F. Costa, Benoit Charlot, Albano C. Meli

**Affiliations:** a PhyMedExp, University of Montpellier, INSERM, CNRS, Montpellier, France; b Biofabics, Porto, Portugal; c IES, University of Montpellier, CNRS, Montpellier, France; d Montpellier Organoid Platform, Biocampus, University of Montpellier, CNRS, INSERM, Montpellier, France

**Keywords:** hiPSC, Microfluidics, Organ-on-chip, Cardiomyocytes, NRs, Neuro-cardiac junction

## Abstract

The neuro-cardiac junction is involved in many pathological conditions in humans, but no model currently allows translational studies to investigate its role. Animal models fail to accurately represent this interaction. This review explores the role of microfluidic technologies in advancing organ-on-chip systems that simulate neuro-cardiac interactions in a controlled environment. By offering precise control over cellular environments, microfluidic platforms significantly enhance the modeling of dynamic cardiac-neural cell interactions. These systems allow the development of more accurate and functional neuro-cardiac junctions, vital for investigating cardiovascular diseases and the neuronal impact in these pathologies. While traditional animal models and co-culture techniques have their merits, they are limited in replicating human-specific physiology. Recent innovations in microfluidics, in combination with human-induced pluripotent stem cell technology, provide more physiologically relevant models and address ethical concerns regarding animal use. This review emphasizes the potential of these advanced microfluidic models in improving disease modeling, drug screening, and therapeutic strategies, ultimately advancing personalized medicine.

## Highlights

• Microfluidic breakthroughs: Reviews cutting-edge organ-on-chip (OOC) platforms that replicate neuro-cardiac junctions (NCJs) using human-induced pluripotent stem cell (hiPSC)-derived neurons and cardiomyocytes, enabling dynamic studies of cardiac-neural interactions.
• Limitations and challenges: Critically evaluates technical barriers, including immature cellular models, specialized seeding techniques (e.g., manual pipetting), and 3D model accessibility, hindering reproducibility and scalability in academic/industrial settings.
• Hybrid system solutions: Proposes hybrid 2D-3D models to balance physiological fidelity with high-throughput drug screening, addressing gaps in standardization and cost-effectiveness.
• Ethical and translational impact: Highlights how microfluidics reduce reliance on animal models, advancing personalized medicine for cardiovascular diseases through patient-specific hiPSC-based platforms.
• Future directions: Advocates for interdisciplinary collaboration, open-source tools, and automation to overcome current limitations, emphasizing applications in disease modeling, drug discovery, and clinical translation.

## Graphical abstract

**Figure:**
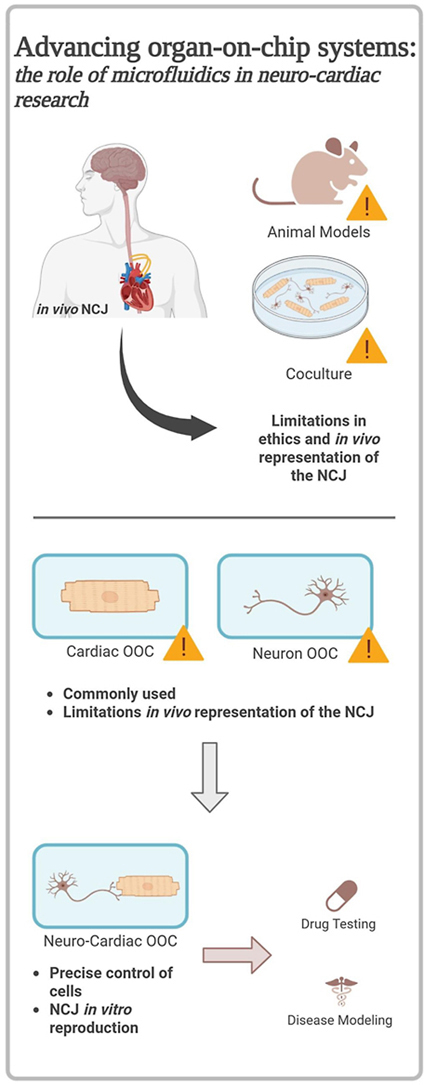

## 1 Introduction

The neuro-cardiac junction is implicated in numerous pathological conditions in humans, yet no existing model enables translational studies to examine its role. It is critical for cardiac and neuronal channelopathies that can lead to sudden death in children, teenagers, and young adults. Additionally, it is involved in common autonomic dysfunctions such as vasovagal syncope, as well as rarer conditions like inappropriate sinus tachycardia and POTS syndrome. Previous studies and derived ablation techniques suggest it may also play a key role in atrial fibrillation (Haegeli and Calkins, 2014). Developing a model of the neuro-cardiac junction will be essential for disease modeling, drug screening, therapeutic strategies, and ultimately, advancing personalized medicine.

The heart and the autonomic nervous system (ANS) have been intimately linked since their embryonic origin, maintaining dynamic interactions throughout life. Damage to either system often necessitates physiological adaptations and, in certain cases, triggers pathological changes. Developing advanced research models for neuro-cardiac studies is vital, as they provide enhanced replication of the intricate *in vivo* interactions and regulatory mechanisms. These models are critical for advancing our understanding, diagnosis, and treatment of cardiovascular and neurological disorders.

Despite their functional interplay, the heart and ANS have distinct embryonic origin. Cardiac cells derive from the mesoderm and differentiate into cardiac progenitors and subsequently into cardiomyocytes (CMs) (Martinez et al., 2015), whereas neural cells originate from the ectoderm. The cardiac wall is a multilayered structure, each layer contributing to its function. The wall comprises three primary layers: the innermost endocardium, the outermost pericardium, and the myocardium, which is the thickest layer and contains contractile CMs (Miller and Gal, 2017). These contractile cells are organized into fibers of varying orientations, anchored to a fibrous skeleton. This structural complexity underpins the heart's ability to autonomously generate electrical impulses, ensuring the synchronized contraction of its chambers. While conduction cells are not contractile, they generate and propagate action potentials (APs), facilitating the heart's rhythmic activity.

CMs, the primary contractile cells of the heart, are characterized by high density of myofibrils. Although present in all cardiac chambers, their properties vary by location. These cells convert electrical signals (APs) into mechanical contractions through excitation-contraction coupling (ECC). This process involves the interplay of ionic movements across membranes and contractile protein activity, leading to sarcomere shortening and cellular contraction.

The regulation of cardiac function is hierarchical, encompassing multiple neural levels that work in concert to meet the body's metabolic demands. This intricate system involves three principal neural networks: the central nervous system (CNS), the extracardiac intrathoracic system, and the intrinsic cardiac nervous system (ICNS) (Fedele and Brand, 2020). The heart employs two primary regulatory feedback loops. A rapid loop, mediated through the intrathoracic system, responds to immediate environmental changes via afferent signals from the heart and blood vessels (Coote, 2013; Dampney et al., 2002; Gebber et al., 1996; Williamson et al., 2006; Zucker et al., 2012). In contrast, a slower feedback loop involves the CNS and integrates systemic physiological responses, particularly during stress or heightened alertness. The brainstem's cardio-regulatory center processes afferent inputs and modulates efferent neuronal output to regulate cardiac activity.

**Fig. 1:**
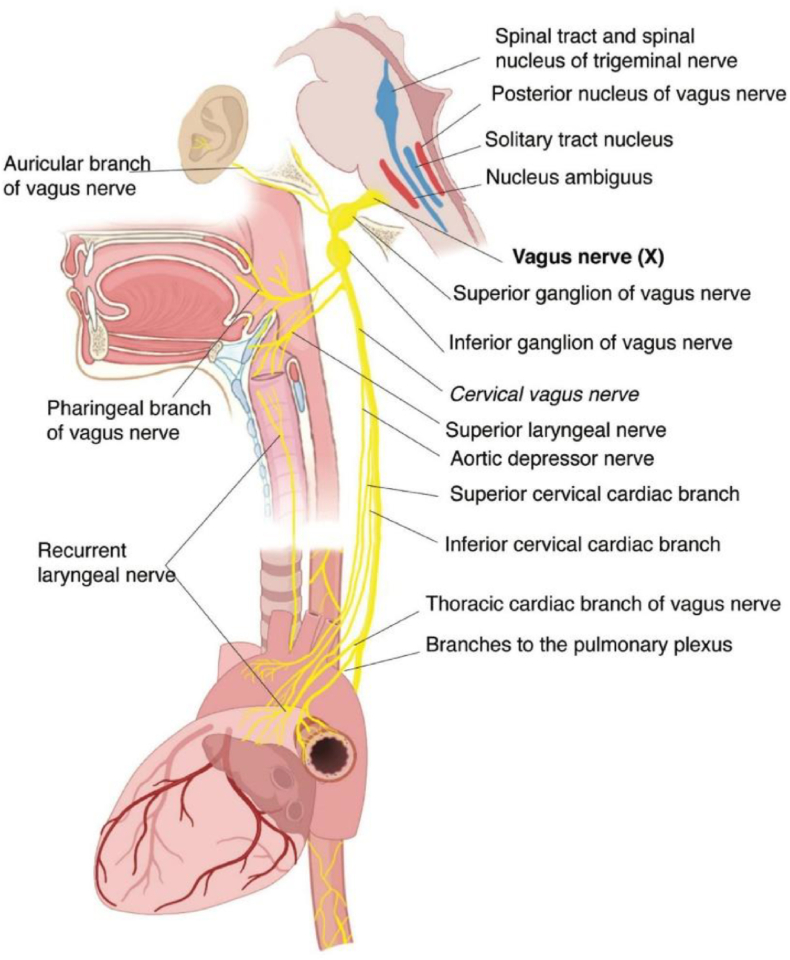
Schematic representation of the cardiac regulation center of the parasympathetic system. Modified from (Ottaviani et al., 2022).

The cardio-regulatory center (Fig. 1) (Ottaviani et al., 2022) integrates inputs from central and peripheral systems and is influenced by higher sensory centers that modulate its activity via GABAergic and glutamatergic neurotransmission (Decavel & Van Den Pol, 1990; Decavel & van den Pol, 1992; K. Zhang and Patel, 1998). Sensory regulation predominates over intrathoracic feedback loops, ensuring cardiac activity aligns with systemic metabolic and circulatory demands (Ardell and Armour, 2016; Fukuda et al., 2015; Kember et al., 2011; Zucker et al., 2012). Afferent signals project to key brain regions, such as the solitary tract nucleus, which further modulate autonomic cardiac control (Cuoco and Fennie, 2016; Kc et al., 2010; Wang et al., 2014).

The ANS meticulously regulates cardiac function to maintain physiological homeostasis across various conditions. For example, during physical exertion, the heart increases blood flow to meet heightened oxygen demands, while activity diminishes during rest.

ANS modulation of cardiac activity involves the interplay of sympathetic and parasympathetic neurons (NRs), which release neurotransmitters at synapses, thereby activating signaling pathways that regulate intracellular systems (Franzoso et al., 2016).

Cardiac development transitions from a hyperplastic phase, marked by cell proliferation, to a hypertrophic phase, characterized by cellular enlargement. The prenatal hyperplastic phase establishes the total CM count, while the postnatal hypertrophic phase determines the heart's final size. Sympathetic nervous system maturity, coinciding with birth, is critical for this developmental progression (Dowell, 1985). Disruptions in noradrenaline (NA) production during this period can impair myocardial growth and, in severe cases, result in fetal demise (Thomas et al., 1995). Studies in rodent models underscore the sympathetic system's role in extending CM proliferation and maturation, which are essential for proper cardiac development (Kreipke and Birren, 2015).

Cardiomyopathies, which disrupt the myocardium's interstitial environment and physical properties, often induce adaptive responses in both cardiac and neuronal tissues. These adaptations involve intracellular signaling cascades leading to structural and functional modifications (Kimura et al., 2012). However, replicating such pathophysiological conditions in models remains a significant challenge due to the limitations of existing cellular and animal models, which often fail to accurately represent human physiological properties.

Recent advancements in cellular engineering, such as co-culture systems and organ-on-chip (OOC) technologies, have provided innovative platforms for studying neuro-cardiac interactions (Häkli et al., 2022; Oh et al., 2016; Prando et al., 2018). Although direct co-culture systems can facilitate physical interactions, they often lack realistic structural organization. Conversely, indirect co-cultures may inhibit necessary cell-cell interactions, such as those required for neuro-cardiac junction formation.

Emerging human-induced pluripotent stem cell (hiPSC) technologies offer promising alternatives. By enabling the differentiation of hiPSC into CMs (hiPSC-CMs) and NRs (hiPSC-NRs), from a unique patient, these models provide enhanced physiological relevance while addressing ethical concerns associated with lab animal use. However, the maturity of hiPSC-derived cells remains a challenge, requiring further optimization to fully replicate adult cellular phenotypes (Karakikes et al., 2015; Karbassi et al., 2020; Lyra-Leite et al., 2022).

2. Animal models to study the neuro-cardiac interactions

Animal models have, historically, been pivotal for understanding physiological and pathological mechanisms, particularly neuro-cardiac interactions. These models provide valuable insights into structural relationships at a macroscopic level, leveraging genetic tools and advanced imaging techniques like rodent heart transparency (Freeman et al., 2014; Rajendran et al., 2019). Recent study on rat hearts has demonstrated the significant role of nerve growth factor, specifically produced by cardiomyocytes, in maintaining the efficacy of the neuro-cardiac junction (NCJ) (Dokshokova et al., 2022).

Animal models present, however, several limitations, both scientific and ethical. While animal models allow the observation of altered autonomic regulations *in vivo* effects on cardiac and neuronal systems, they often fail to capture the intricate cellular-level interactions inherent to neuro-cardiac junctions. These junctions involve both physical and chemical signaling, which are inadequately replicated when cardiomyocytes are exposed to adrenergic agonists alone. Results from animal models may not always directly translate to humans due to physiological differences. Among these differences, it is particularly interesting to note that the autonomic nervous system regulates cardiac function, with a greater impact of the nervous-sympathetic system in rodents than in humans, resulting in a heart rate 10 times higher in rodents than in humans. Interspecies genetic and physiological differences hinder the accurate modeling of human pathologies and responses to therapeutic interventions.

Ethical concerns further complicate the use of animal models. Researchers must weigh the scientific necessity of such experiments against the potential for animal suffering and bioethical considerations surrounding welfare (Kiani et al., 2022). Despite these challenges, animal models remain indispensable, underpinning numerous medical advancements in pharmacology in areas such as vaccine development, cancer treatment, and also in the understanding of fundamental biological processes. Nonetheless, there is an ongoing push within the scientific community to refine, reduce, and replace animal models (3Rs) with alternative methods that uphold ethical and scientific rigor (B. Zhang et al., 2018).

3. Co-culture techniques with animal cells: advantages and limitations

Co-culture experiments have been instrumental in studying neuro-cardiac interactions, employing either direct or indirect approaches. In indirect co-culture setups, cells are grown in proximity without physical contact, while direct co-culture involves seeding different cell types within the same environment to enable physical interaction.

Past studies using co-cultures have demonstrated beneficial effects on both cardiac and neuronal cells. For example, CMs and NRs co-cultured on coverslips revealed specialized post-junctional zones on CMs where neurites made synaptic-like contacts (Häkli et al., 2022; Prando et al., 2018; Shcherbakova et al., 2007; Winbo et al., 2020). Similarly, indirect co-cultures using microfluidic chips with microtunnels have facilitated axonal growth toward cardiac cells, enabling neuro-cardiac junction formation (Oiwa et al., 2016; Sakai et al., 2017; Takeuchi et al., 2011).

However, these approaches are not without limitations. Direct co-culture methods fail to mimic the physiological structural organization, as NRs and their axons share the same environment, unlike *in vivo* conditions. Indirect co-cultures, while allowing compartmentalization, often hinder essential physical interactions, thereby limiting the development of authentic neuro-cardiac junctions. Co-culture without separate compartments lacks rigorous and accurate control of each cellular environment.

The advent of human-induced pluripotent stem cell (hiPSC) technology has shifted the paradigm, enabling part-human and fully human co-culture models. These systems improve physiological relevance and reduce the ethical concerns associated with animal models (Bernardin et al., 2022; Kirino et al., 2018; Kowalski et al., 2022; Oh et al., 2016; Winbo et al., 2020).

4. Human induced pluripotent stem cells

In 2006, Yamanaka and his team identified four key genes essential for achieving pluripotency and embryonic stem cell (ESC) renewal (Takahashi and Yamanaka, 2006). This landmark discovery led to the generation of human induced pluripotent stem cells (hiPSC) in 2007 (Takahashi et al., 2007). The development of hiPSC marked a major milestone in biomedical research, introducing versatile and non-invasive tools that preserve a patient's genetic background while enabling the generation of various cell types. Additionally, hiPSC address several limitations inherent in animal models, including ethical concerns and the inability to fully replicate human pathophysiology.

Reprogramming hiPSC can be achieved using a range of somatic cells, and significant advancements have been made since the initial techniques were developed in 2007. Two primary approaches exist for hiPSC reprogramming: integrative and non-integrative methods. The integrative approach, which involves introducing transcription genes via retroviral or lentiviral vectors (Takahashi et al., 2007), was the first method to be developed. However, it poses risks such as insertional mutagenesis and the potential reactivation of transcription genes, which can compromise cell proliferation and differentiation. In contrast, the non-integrative approach, a more recent development, avoids genomic integration by employing vectors derived from the Sendai virus (FUSAKI et al., 2009), Epstein-Barr virus episomes (C. M. Chang et al., 2009), or synthetic mRNAs (Warren et al., 2010).

hiPSC possess the remarkable ability to differentiate into cell types originating from any of the three embryonic germ layers, including cardiomyocytes (derived from the mesoderm) and NRs (derived from the ectoderm).

### 1.1 Cardiac differ*entia*tion

The first successful cardiac differentiation of ESCs was achieved in 2001 using embryoid bodies - 3D aggregates of human ESCs cultured in suspension with DMEM-KO and 20 % fetal bovine serum (FBS). This process led to the spontaneous differentiation of cardiomyocytes, albeit with an efficiency of only around 8 % (Kehat et al., 2001). In 2003, Mummery and colleagues introduced a novel method involving the co-culture of human ESCs with mice visceral endodermal cells (Mummery et al., 2003). Despite its low yield, this method identified critical factors for ventricular differentiation, such as BMPs, FGFs, and inhibitors of the Wnt/ß-catenin pathway. These findings spurred the development of advanced cardiac differentiation protocols in both 2D (Laflamme et al., 2007) and 3D formats (Burridge et al., 2007). Laflamme's study in 2007 was the first to highlight the efficacy of the RPMI/B27 combination, while subsequent work revealed the inhibitory effect of insulin during cardiogenesis (X. Q. Xu et al., 2008). In 2008, Zhang and colleagues successfully generated cardiomyocytes from hiPSC, demonstrating that these cells share characteristics with those derived from ESCs (J. Zhang et al., 2009). Later studies aimed to optimize costs and efficiency, culminating in protocols such as GiWi, which utilized smaller molecules for monolayer differentiation (Lian et al., 2012, 2013). In 2014, Burridge's team introduced the CDM3 protocol, achieving yields of approximately 90 % with chemically defined media (Burridge et al., 2014). We have demonstrated the pertinence of AggreWell plates to aggregate a specific number of single cells results in a large number of uniform 3D cardiac embryoid bodies in size and shape (Pesl et al., 2014), offering a dependable micromechanical cellular biosensor capable of continuous testing throughout the day (Pesl et al., 2016).

Despite these advancements, a persistent challenge remains: the immaturity of hiPSC-derived cardiomyocytes, which retain fetal-like structural and functional properties. Compared to adult cardiomyocytes, these cells exhibit several key differences (Karakikes et al., 2015; Karbassi et al., 2020; Lyra-Leite et al., 2022). Structurally, they display random shapes, disorganized sarcomeres, and smaller volumes, with underdeveloped sarcoplasmic reticula (SR) lacking T-tubules. This underdevelopment results in poor calcium handling and suboptimal excitation-contraction coupling, leading to reduced contractile force. Electrophysiologically, these cells have less negative resting potentials and exhibit shorter action potential durations and amplitudes, which can favor automatic activity, observable even on cells with a ventricular profile. Furthermore, their metabolic profile is distinct, relying on glycolysis rather than the oxidative metabolism characteristic of mature cardiomyocytes (Karbassi et al., 2020).

### 1.2 Neuronal differentiation

Stem cell-derived NRs similarly offer transformative potential for the study of neurological diseases. These cells reflect the genetic background of patient populations, enabling disease modeling and drug screening. Over recent years, numerous protocols have been developed for generating specific neuronal subtypes from hiPSC. These protocols generally involve three main stages: induction, differentiation, and maturation.

In 2016, Oh and colleagues reported the first successful differentiation of hiPSC into sympathetic NRs using techniques such as homologous recombination and CRISPR/Cas9 (Oh et al., 2016). These sympathetic NRs were capable of releasing noradrenalin (NA), modulating the heart rate of co-cultured cardiomyocytes. However, functional coupling between NRs and cardiomyocytes was not achieved. In 2020, two independent studies by Takayama and Winbo introduced chemical differentiation protocols for generating sympathetic NRs using embryoid bodies and 2D monolayer cultures, respectively (Takayama et al., 2020; Winbo et al., 2020). Both groups managed to obtain ANS NRs and showed that NRs derived from hiPSC express markers specific to autonomic NRs (Takayama et al., 2020; Winbo et al., 2020). Furthermore, both groups demonstrated that stimulation of hiPSC-NRs was sufficient to modulate the chronotropic activity of cardiomyocytes by increasing the heart rate (Takayama et al., 2020; Winbo et al., 2020). On one hand, Winbo's study was notable for its electrophysiological characterization of sympathetic NRs, which represents a critical contribution, alongside the demonstration of cholinergic receptor expression. The use of a monolayer configuration in their protocol offered a practical advantage due to its ease of implementation (Winbo et al., 2020). On the other hand, Takayama's work highlighted the capacity to derive both sympathetic and parasympathetic NRs from a single neuronal induction process. Their findings further underscored the functional maturity of these NRs, as evidenced by their response to pharmacological agonists like nicotine, while showing no response to menthol, a compound that selectively activates sensory NRs (Takayama et al., 2020).

A recent study successfully utilized Winbo's protocol to derive sympathetic NRs from human-induced pluripotent stem cells (hiPSC). This work further validated the chemical differentiation protocol, producing hiPSC-derived sympathetic NRs characterized by round somas and extensive axonal growth. These NRs exhibited normal behavior in response to receptor activation and voltage-induced calcium regulation. Together with the findings of Winbo's group, these results suggest that differentiating sympathetic NRs from hiPSC lines reliably generates functional NRs capable of responding to physiological stimuli (Li et al., 2023).

## 2 Microfluidic technology for biomedical applications

Microfluidic technologies, especially those based on polydimethylsiloxane (PDMS), are particularly well-suited to cell culture. In addition to its ease of microfabrication by molding and replication technique on a photosensitive resin mold, and its biocompatibility, this material exhibits a certain degree of porosity which allows the diffusion of Oxygen (O_2_) and Carbon dioxide CO_2_, and thus participates in cellular respiration, which is necessary to keep cell cultures alive over the long term. In addition, its visible transparency and lack of autofluorescence make it a perfect tool to study cell behavior under the microscope. Microfluidic technologies therefore make it possible to create a culture environment with cell-scale chambers and channels, reconstructing an environment that can mimic *in vivo* and its structures. These include, for example, micro-constriction structures for studying cell migration mechanisms under chemokine gradients (Ren et al., 2022), but also compartmentalized circuits which, thanks to a network of microchannels whose dimensions are smaller than cell bodies, recreate guidance channels enabling neurites to engage in them and separate cell populations, thus recreating neuronal junctions (Moutaux et al., 2018) such as the cortico-striatal junctions (Virlogeux et al., 2018) at work in Huntington's disease, for example. These compartmentalized circuits can also be used to recreate motor NR-muscle (Duc et al., 2021) or NR-skin junctions (Risueño et al., 2021).

### 2.1 The OOC and its principle

The OOC concept consists in using microfluidic technologies to construct in vitro, in microfluidic circuits, assemblies of one or more cell types mimicking a particular mechanobiological environment that favors a cellular organization found *in vivo*. Examples include gut on a chip, where epithelial cells are cultured on microstructured scaffolds modelling the crypts and villi of the intestine (Verhulsel et al., 2023), or channels, where microporous membranes are used to separate compartments or microchannels containing a covering of endothelial cells to mimic the blood-brain barrier (Hajal et al., 2022), and to study the passage of particular molecules across it. Numerous research projects are focusing on the development of OOC or microphysiological systems (MPS) in the fields of neuroscience, diabetology, cancerology and cardiology.

However, with the advancement of organoids, spheroids, and assembloids, biological models are becoming increasingly complex. They are transitioning from two-dimensional cell layers to large, spheroidal cell clusters up to 1 mm in size. This shift presents new challenges in developing microfluidic circuits specifically designed for OOC systems based on these spheroids (Hetzel et al., 2023). This concerns not only the maintenance and cultivation of these cell clusters, but also electrophysiology techniques (Phouphetlinthong et al., 2023), with the ever-important challenge of vascularizing these organoids (Quintard et al., 2024).

### 2.2 The cardiac OOC

The development of cardiac OOC technology began in the early 1990s with the introduction of tissue scaffolds. These scaffolds, typically 2D substrates made from polydimethylsiloxane (PDMS), provided a surface for cell attachment, growth, and survival. By 2004, it was recognized that electrical stimulation was crucial for creating cardiac tissues that more closely replicated the *in vivo* physiology of the heart. Efforts to improve these models led to a transition from 2D to 3D scaffolds in the late 2000s, enabling the formation of more complex tissue structures. By 2010, the combination of electrical and mechanical stimulation further enhanced tissue maturation. By 2018, systems incorporating both types of stimuli became widely available. The ability to generate more mature tissues shifted the focus toward real-time monitoring of tissue functions. One commonly used technology was the microelectrode array (MEA), which allowed researchers to record electrophysiological activity from cardiac tissues, providing real-time data on tissue health and functionality. Simultaneously, advances in materials science led to the development of more biocompatible and flexible materials that interacted more naturally with cardiac cells, thereby supporting long-term tissue growth (F. Xu et al., 2024).

The cardiac OOC models aim to partially replicate the intricate microenvironment of the human heart, which depends on its mechanical, electrical, and structural properties for coordinated function. Key features such as anisotropic tissue structures, electrophysiological activity, and rhythmic contraction are essential for accurately mimicking native heart tissue (F. Xu et al., 2024). These models consist of three primary domains: scaffolds, stimulation systems, and sensors. Scaffolds provide structural support for cell adhesion, growth, and maturation while influencing cellular behavior through their mechanical properties (F. Xu et al., 2024). Real-time monitoring, enabled by integrated sensors, is critical for assessing tissue health, maturity, and responses to external stimuli or drugs. Mechanical and electrical sensors track essential physiological parameters such as contractile force and electrical activity, facilitating continuous, non-invasive evaluation. This functionality is particularly important for high-throughput drug screening and long-term cardiac tissue monitoring (F. Xu et al., 2024).

Two main types of scaffolds are used in cardiac OOC devices: 2D and 3D scaffolds. The stiffness of 2D scaffolds is typically controlled using flexible materials or micro/nano-structured designs, such as micropillar arrays, grooves, or fibrous networks (Yu et al., 2021). In contrast, 3D scaffolds provide a volumetric growth environment that supports cell-cell and cell-extracellular matrix (ECM) interactions, enhancing physiological relevance (Yu et al., 2021). These 3D scaffolds can be further classified as porous or bioprinted. Porous scaffolds, often made from hydrogels, are cured using ultraviolet light (B. Zhang et al., 2016). Electrospinning techniques can also be employed to produce fibrous scaffolds with defined porosity (H. Chang et al., 2022; Wu et al., 2017). Alternatively, bioprinting allows for the creation of volumetric scaffolds by printing hydrogels infused with cardiac cells, a method particularly suitable for thick cardiac tissue engineering (Y. S. Zhang et al., 2016).

Stimulation components play a crucial role in promoting cardiac tissue maturation by incorporating both electrical and mechanical cues. For electrical stimulation, factors such as electrode biocompatibility, uniformity of the electric field, and alignment of the field with tissue growth must be carefully considered. Electrical pulses should also remain within the non-Faraday response voltage range (F. Xu et al., 2024). Electrical stimulation can be delivered via rod electrodes or 2D patterned electrodes. Rod electrodes, valued for their simplicity and reliability, can be fabricated from materials such as carbon, stainless steel, platinum, or conductive biomaterials (López-Canosa et al., 2021; Pavesi et al., 2015; Radisic et al., 2004; Visone et al., 2018; F. Zhang et al., 2021). In comparison, 2D patterned electrodes, often made from gold films, are versatile and can be combined with additional sensors to enhance their functionality. However, these electrodes are less effective for thick tissue models (Wu et al., 2017; B. Zhang et al., 2016). Mechanical stimulation employs passive or active structures. Passive structures, such as flexible frames or cantilevers, provide reactive forces that promote tissue alignment and maturation (Liu et al., 2020; Ronaldson-Bouchard et al., 2018; D. Zhang et al., 2013). Active structures, on the other hand, utilize stretchable membranes that dynamically deform, simulating physiological mechanical stimuli (He et al., 2020; Huh et al., 2013; Kim et al., 2012; Novak et al., 2020; Zheng et al., 2012).

Sensors are integral to cardiac OOC, enabling real-time monitoring of cardiac tissue functionality. These include force sensors, which measure tissue contraction by translating deformation into measurable signals, and electrophysiological sensors, which track electrical changes during contraction. Force sensors can be designed for single-cell, thin-tissue, or thick-tissue applications. For thin tissues, technologies such as the CellDrum, a thin-film PDMS membrane and cantilevers, constructed via microfabrication or 3D printing, are commonly used. These devices detect tissue contractility by measuring mechanical deformation or bending caused by cellular contraction (Goßmann et al., 2016; Grosberg et al., 2011; Lind et al., 2017; Lind et al., 2017; Linder et al., 2010; McCain et al., 2014). For thicker tissues, flexible columns or beams measure deflections correlated with tissue maturity and function (F. Xu et al., 2024).

MEA provides non-invasive measurements of extracellular field and action potentials through arrays in direct contact with the tissue (Kussauer et al., 2019). MEA is categorized into 2D and 3D configurations, depending on the tissue type. For thin tissues, 2D MEA, typically made from noble metals such as gold and platinum or conductive polymers and mounted on rigid substrates, are employed (Alassaf et al., 2020; J. S. Park et al., 2019). High-density and high-resolution electrodes in 2D MEA enable precise signal detection (L. Xu et al., 2021). For thicker tissues, 3D MEA offers superior spatial resolution by capturing signals in a three-dimensional space. These devices use mechanically guided substrate deformation to position electrodes within the 3D environment, facilitating comprehensive electrophysiological analysis (Bo et al., 2023; Cho et al., 2021).

### 2.3 The neuronal OOC

Although the concept of OOC technology was first introduced in 2011, in the field of neuroscience, the idea of “on-chip” technology was established much earlier. However, the specific term "neuron-on-chip" was first coined in 2015. Neuronal OOC systems are designed to facilitate the formation of interactive neuronal networks, rather than highly structured tissues. These systems enable the study of spatial-temporal resolution, cellular arrangement, differentiation, neurotransmission, and circuit control, making them invaluable for neuroscientific research (Buentello et al., 2024).

The architecture of neuronal OOC can vary based on the intended application. The earliest design, introduced by Campenot in 1977, featured two separate microcompartments connected by microchannels (Campenot, 1977). This foundational design inspired numerous iterations, most of which incorporated microchambers linked by microchannels. These systems typically included two or three compartments, allowing for the separation of neuronal soma from axons and integrating central perfusion channels for pharmacological testing (J. W. Park et al., 2006; Taylor et al., 2003, 2010). Other variations included chips with concave microwells that utilized interstitial flow (J. Park et al., 2015). In parallel, technological advancements were achieved by commercial ventures, such as Netri, MicroBrain Biotech or Minetas. Minetas developed the OrganoPlate, an innovative 3D microfluidic titer plate system (Spijkers et al., 2021; Wevers et al., 2016). Another variant was fabricated from PDMS and included three interconnected microchambers to culture distinct cell types simultaneously (Dauth et al., 2017). More recently, a 3D device was introduced with two concentric microchambers connected by migration channels, enabling cell migration and interaction in a controlled environment (J. Park et al., 2018).

Design customization is not limited to the structural layout; geometry, patterning, and biomaterials can also be adjusted depending on the biological models being used (Table 1) and the type of studies being conducted (Table 2). Fine-tuning the neuronal microenvironment improves physiological modeling by accounting for extracellular components, geometric constraints, and regional seeding. These variables are essential for accurately studying neuronal behavior in neuronal OOC systems. The geometry and patterning of the chip significantly influence neuronal processes. For example, ridges, grooves, nodes, and channels can modulate adhesion, morphology, migration, proliferation, and differentiation. Microcontact-printed patterns offer precise control over neuronal networks by directing synaptic formation. Porosity and pore size affect neuronal anchorage and migration patterns, while grooves and channels can guide cell growth and fiber alignment (Buentello et al., 2024).

**Table 1:** Biological models used in neuronal OOC (Buentello et al., 2024).

Primary Cells	Stem Cells	Immortalized Cell Lines
C. elegans	D3 mES	Glioblastoma cell lines
Chick forebrain NRs	H9 hESCs	H4 neuroglioma
Chick dorsal root ganglia	hiPSC-NRs	Human teratocarcinoma cell lines
Drosophila	ReNcell VM cells to NR	Immortalized murine brain endothelial cells
*Lymnaea stagnalis* ganglion	hNESC	
Mouse cortical	Mouse embryonic Stem Cells	
Rat cerebral cortical/hippocampal	Human ESC	
Rat dorsal ganglia	Neural Stem Cells (NSC)	
Rat hippocampal slices	Mouse cortex NSC	
*Aplysia californica*	Rat-derived Neural Progenitor Cells	
Zebrafish	SH-SY5Y

**Table 2:** Types of studies performed on neuronal OOC (Buentello et al., 2024).

Types of Studies
Chemical Stimulation
Mechanical Stimulation
Neural Network reconfiguration
Neurite Growth and Manipulation
Axonal Growth and Manipulation
Spheroid Migration
Auditory Stimulation
Topological Studies
Cell Trapping
Optogenetics
Axonal Transport
Disease Modelling
Differentiation
Compartmentalization
Axonal Injury
Pharmacological Testing
Neuroinflammation
Neuron-glial interactions
Electrophysiology
Organoids

Incorporating ECM components into neuronal OOC further enhances neuronal anchorage, survival, and differentiation in both 2D and 3D cultures. Commonly used ECM biomaterials include laminin, fibronectin, collagen, polylysine (PLL/PDL), and poly-L-ornithine (PLO), although Matrigel remains the most widely employed (Buentello et al., 2024). The choice between 2D and 3D culture determines the selection of biomaterials and necessitates consideration of ECM rigidity, which profoundly affects neuronal growth and network formation. A rigid ECM promotes glial cell development, whereas a less rigid ECM favors neuronal differentiation.

While 2D neuronal cultures have yielded significant insights into intracellular molecular mechanisms, they provide a poor representation of *in vivo* neuronal growth. Aberrant genetic cascades, abnormal migration, faulty attachment mechanisms, and increased apoptosis via Anoikis have been observed in 2D cultures. Conversely, 3D cultures, often using hydrogels, more accurately replicate the mechanical properties of neuronal tissues. The choice of biomaterials in 3D cultures influences neuronal outcomes. For instance, agarose-alginate mixtures support cortical layer patterning, gelatin-based scaffolds enhance adhesion during perfusion, and collagen I scaffolds facilitate growth factor diffusion and cell migration. Capgel scaffolds promote axon bundling, while synthetic materials such as glass microbeads and PDMS micro-lattices offer additional options for creating specialized environments (Buentello et al., 2024).

Neuronal OOC features numerous tunable elements, enabling a wide range of applications, including axon compartmentalization, co-culture systems (Table 3), flow and gradient studies, electrophysiological analyses, and disease modeling.

**Table 3:** Applications of neuronal OOC for co-culture (Buentello et al., 2024).

Type of neuro-on-chip	Applications
Microchannels	Isolate hippocampal NRs from other NRs or astrocytes
OrganoPlate	Study neuron and glial interaction after neurotoxic insults
Tricultures	Study different metabolic needs and firing rates in cortex, hippocampal and glial cells
2-chamber	Studies on neuro-muscular junctions

### 2.4 The neuro-cardiac OOC

In recent years, there has been a surge in the development of microfluidic devices designed to study the neuro-cardiac junction (Table 4). These devices typically feature microchambers for seeding neuronal cells and cardiomyocytes, and microchannels that connect the compartments. The microchannels are sufficiently wide to accommodate axons but narrow enough to prevent the somas of the NRs from passing through. This design aims to recreate an *in vivo*-like environment, where the neuronal somas do not directly interact with cardiomyocytes.

**Table 4:** List of neuro-cardiac OOC devices described in literature.

Cell Type	OOC Design	Materials	Functional Readout	Applications	Ref
Rat SCG NRs	Microchamber with sixteen microcompartments and eight microconduits	PDMS-based	Electrical	Electrophysiological studies	Takeuchi et al. (2011)
Rat ventricular myocytes VMs					
Rat SCG NRs	Two compartments connected by microchannels	PDMS-based	Electrical	Electrophysiological studies	Takeuchi et al. (2012)
Pluripotent Murine Embryonal Cell derived EBs					
Rat SCG NRs	GelPins that use surface tension of liquids for spatial separation of cells	Polyethylene terephthalate (PET)	Electrical	Electrophysiological studies	Soucy et al. (2020)
Rat Cardiomyocytes					
hiPSC-derived NRs	Two neuronal compartments on either side and a central co-culture compartment interconnected by microtunnels	PDMS-based	Electrical Biochemical	Electrophysiological studies Biochemical studies (RNA extraction, immunocytochemistry, morphology analysis)	Soucy et al. (2020)
hiPSC-derived cardiomyocytes				Microscopy	
Rat-derived NRs hiPSC-derived cardiomyocytes	Two chambers connected by asymmetrical microchannels	Epoxy-based negative photoresists	Electrical	Electrophysiological studies	Bernardin et al. (2022)
hiPSC-derived autonomic NRs				Calcium handling experiments	
hiPSC-derived cardiomyocytes				Pharmacological studies	
				Microscopy	

**Fig. 2:**
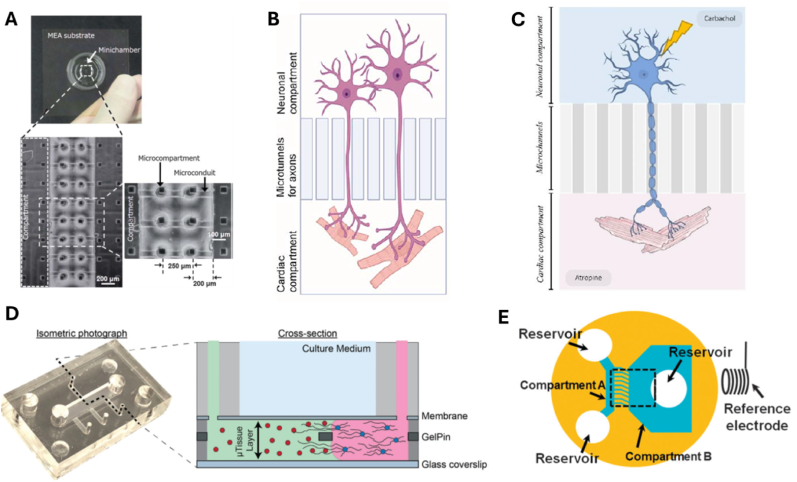
Neuro-cardio OOC designs. (A) First interaction of the neuro-cardio OOC (Takeuchi et al., 2011). (B) Schematic of the 3D3C chip (Häkli et al., 2022). (C) Schematic of the neuro-cardio OOC with asymmetrical channels (Bernardin et al., 2022). (D) Neuro-cardiac OOC using GelPin technology (Soucy et al., 2020). (E) Two chambers of the neuro-cardiac OOC (Takeuchi et al., 2012).

The first use of microfluidic devices to simulate the interaction between NRs and cardiomyocytes was reported in 2011 by Takeuchi et al., who developed a PDMS-based co-culture device consisting of MEA and a microchamber (Fig. 2A). This chamber contained sixteen microcompartments for cell seeding and eight microconduits to support neurite growth for cardiac innervation. For testing, the authors used superior cervical ganglia (SCG) NRs and ventricular myocytes (VMs) from rats. The SCG NRs and VMs adhered to their respective compartments and were physically separate, with only the SCG neurites connecting to the VMs. Spontaneous contraction of the VMs was observed, and electrical stimulation of the SCG NRs evoked a response that could be recorded by the electrodes, regardless of pulse frequency or number. Additionally, neuronal responses were only recorded from electrodes directly under the stimulated NRs. Importantly, electrical stimulation of the NRs led to a change in the beat rate of the VMs, suggesting the formation of a synaptic pathway between the SCG NRs and VMs. A limitation of this device was the need for special glass pipettes to ensure proper seeding of the cells in the microcompartments (Takeuchi et al., 2011).

Building on this concept, the same authors developed an improved version of the device in 2012. This iteration maintained the MEA substrate but featured a different chamber design (Fig. 2E), both components being fabricated using photolithography techniques. The chamber consisted of two compartments (A and B) connected by microchannels, which allowed only neurite passage, while preventing the soma of the NRs from crossing into the other compartment. In compartment A, where SCG NRs were seeded, two reservoirs and sixteen pockets were integrated to place NRs over the electrodes. Unlike the earlier device, EBs derived from pluripotent murine embryonal cells were used in place of rat VMs, allowing the study of the device in a 3D environment. In this new device, the NRs adhered to the pockets in compartment A, while the EBs remained in compartment B, with only the neurites passing through the microchannels. Electrical stimulation of SCG NRs again led to a change in the beating rate of the cardiomyocytes. The key advantage of this design over the previous one was the elimination of the need for special glass pipettes, as compartment A itself served as a flow tunnel for cell seeding (Takeuchi et al., 2012).

Subsequent designs from other research groups continued to explore microfluidic devices and OOC aimed at replicating the *in vivo* neuro-cardiac junction. In 2020, Soucy et al. introduced a novel microfluidic device that departed from the previously described designs by using GelPins, thin layers of polyethylene terephthalate (PET), to leverage the surface tension of liquids for spatial separation of cells cultured in GeIMA (Fig. 2D). Cardiomyocytes were loaded with the hydrogel into the device, which was then crosslinked using visible light. Following this, sympathetic NRs were loaded into the device. The GelPin structure allowed the hydrogel to remain in the designated compartments, facilitating the creation of multiple device layouts, as its architecture could be adjusted in both the x-y plane and the z-direction. Additionally, the absence of microchannels and microchambers enabled a more efficient use of the surface area and facilitated the development of more complex 3D geometric patterns. The device was placed on a commercial MEA for real-time data collection and electrophysiological analysis (Soucy et al., 2020).

In 2022, Häkli et al. developed a new neuro-cardiac OOC, named the 3D3C, which consists of two PDMS-based sections: a cell culture section mounted on a glass coverslip and a medium chamber section placed on top of the cell culture section (Fig. 2B). The cell culture section includes two neuronal compartments on either side and a central co-culture compartment. These compartments are interconnected by 40 microtunnels that separate the axons from the somas. The medium chamber section, located above the cell culture section, includes three distinct chambers that allow for the use of cell-specific media for each cell type. The authors tested the device with hiPSC-derived NRs and cardiomyocytes. The design enabled the successful isolation of axons and the formation of functional connections between the NRs and cardiomyocytes, as confirmed by the use of synaptic markers. Additionally, the design permitted the extraction of NRs or cardiomyocytes without cross-contamination. This chip allowed electrical stimulation of the NRs to assess their impact on cardiomyocytes and supported various analytical techniques, including RNA extraction for gene expression studies, immunocytochemistry for protein expression and morphology analysis, and functionality studies using video microscopy (Häkli et al., 2022).

Also in 2022, we and MicroBrain Biotech have reported another microfluidic device for neuro-cardiac OOC (Fig. 2C). This device incorporated microfluidic components fabricated using standard molding techniques with epoxy-based negative photoresists and a proprietary chip design. The microfluidic component consisted of two chambers, one for NRs and the other for cardiomyocytes, connected by asymmetrical microchannels, where the opening of the microchannel differed on each side. Rat-derived NRs were seeded into the chamber with the larger microchannel opening, while hiPSC-derived cardiomyocytes were seeded into the other chamber. This design aimed to promote the innervation of the cardiomyocyte chamber by neuronal projections. The microfluidic architecture enabled fluidic isolation between the chambers, which facilitated the targeted application of pharmacological agents to the desired compartment. Immunofluorescence experiments confirmed the development of neuro-cardiac junctions and synapse-like junctions, and the device supported electrophysiological studies and calcium handling experiments. The device was further tested using both hiPSC-derived autonomic NRs and hiPSC-derived cardiomyocytes, yielding results consistent with those obtained from the initial combination of rat NRs and hiPSC-derived cardiomyocytes (Bernardin et al., 2022).

### 2.5 Industry and commercially available OOC

The development of platforms to support the study of the neuro-cardiac junction is an emerging research field. Currently, there are no widely commercialized platforms developed solely for this purpose. However, several companies offer flexible microphysiological systems that could be adapted for this application.

NETRI has developed the NeuroFluidics™ platform, designed for studying neuronal networks in compartmentalized environments. This platform supports directional connectivity and axonal guidance, which are crucial for modeling synaptic interactions between NRs and cardiomyocytes. Additionally, these chips are compatible with automated workflows and high-throughput analysis (NETRI, 2025).

Emulate Inc. offers the Chip-S1®, a stretchable, dual-channel microfluidic chip capable of simulating physiological stretch and fluid shear, two essential factors for mimicking cardiac environments. Although primarily marketed for lung, intestine, and liver models, the Chip-S1® supports co-culture formats and could potentially be adapted to study neuron-cardiomyocyte interactions by leveraging its mechanical actuation and perfusion capabilities. Its flexible membrane allows for dynamic cell interaction across compartments, an essential feature for mimicking the sympathetic regulation of cardiac function (Emulate Inc., 2025).

CN Bio provides the PhysioMimix® OOC platform, known for its ability to sustain organ-specific tissue cultures under continuous perfusion. While it is primarily used for liver and multi-organ systems, its modular design could allow for the integration of custom tissue types, including neural and cardiac tissues, under controlled physiological conditions (CN Bio, 2025).

Mimetas offers the OrganoPlate®, a high-throughput 3D culture platform using phaseguide technology to control fluid flow without pumps. Originally developed for barrier and organoid models, the system's layout could be adapted for studies involving electrically active tissues. With modifications such as integrating microelectrode arrays, the OrganoPlate® could serve as a platform for exploring electrophysiological coupling between NRs and cardiomyocytes (Mimetas, 2025).

TissUse provides the HUMIMIC platform, which supports multi-organ co-culture and can be leveraged for complex inter-organ communication models. The system allows for dynamic perfusion between up to four organ compartments, making it suitable for building a neuro-cardiac axis model by integrating neural and cardiac tissues into a physiologically relevant circuit (TissUse, 2025).

Another notable entry is the Axosim platform. While Axosim primarily focuses on neuro-focused microphysiological systems such as the Nerve-on-Chip®, there is potential to combine their neural modeling technology with cardiac cell platforms through collaboration or hybrid model development (Axosim, 2025). Although not commercially marketed for neuro-cardiac studies, this approach opens the door to assembling modular systems that mimic long-range interactions such as vagal and sympathetic cardiac control.

Collectively, these platforms form a flexible technological ecosystem. While none are explicitly marketed for the neuro-cardiac junction, their underlying capabilitiesco-culture compatibility, controlled fluid flow, mechanical stimulation, and electrophysiological monitoringmake them well-suited for adaptation to study this intricate physiological interface.

Despite the high customizability and technical capabilities of existing OOC platforms, there remains a notable gap in dedicated neuro-cardiac junction systems in both academia and the commercial sector. Most current technologies have been developed with a focus on single-tissue models or well-established organ pairs such as gut-liver or lung-endothelium, leaving the neuro-cardiac axis relatively underexplored. Consequently, researchers seeking to study the bidirectional communication between NRs and cardiomyocytes often need to significantly adapt existing platforms or design bespoke systems from scratch. This lack of off-the-shelf solutions limits accessibility, scalability, and standardization in the field, highlighting a critical opportunity for innovation in both academic research and industry product development.

## 3 Discussion

In recent years, the development of microfluidic platforms has significantly enhanced the ability of researchers to create advanced in vitro models across various fields, including cardiac and neuronal research. These platforms have facilitated the generation of more replicable and physiologically relevant models to mimic *in vivo* conditions. The integration of human-induced pluripotent stem cells (hiPSC) has further expanded the possibilities for generating accurate and functional in vitro replicas. However, within the realm of neuro-cardiac research, particularly concerning neuro-cardiac junctions (NCJs), the development of such models and devices remains noticeably inadequate.

Neuro-cardiac interactions encompass multiple layers of complexity that current in vitro models generally fail to fully recapitulate. These interactions involve both rapid electrical signalling and slower neuromodulatory processes mediated by second messengers, some dynamic features that remain challenging to capture with existing biosensors and imaging technologies (Zucker et al., 2012). Another critical aspect is the intricate vascular and immune microenvironment present at the neuro-cardiac junction, which is largely omitted in most in vitro systems. This omission significantly limits their applicability in studying inflammation-related pathologies or evaluating drug responses (Quintard et al., 2024).

Beyond biological complexity, neuronal-cardiac co-cultures often exhibit functional degradation within a few days in vitro, underscoring the difficulty in maintaining long-term viability and physiological function, a major technical challenge (Prando et al., 2018). Furthermore, despite the widespread use of conventional readouts such as MEAs and calcium imaging, these techniques provide only limited insight into key phenomena such as synaptic plasticity, neurotransmitter release kinetics, and network-level dynamics (L. Xu et al., 2021). Collectively, these limitations suggest that current in vitro platforms may be insufficient for accurately modelling the full physiological context of neuro-cardiac regulation.

The lack of studies dedicated to organ-on-a-chip (OOC) technologies for NCJ research is striking. Since the field began exploring this area in 2011, only a handful of publications have described the design or application of OOCs specifically for neuro-cardiac interactions. Several factors might have contributed to the slow progress in this field. Co-culturing hiPSC-derived NRs and CMs presents significant challenges due to their distinct requirements for growth factors, oxygen levels, and maturation kinetics, which collectively hinder long-term viability and functional stability (Karbassi et al., 2020; Mummery et al., 2003). In addition, unlike more established OOC systems involving other cell types, neuro-cardiac platforms are seldom available as commercial off-the-shelf solutions. As a result, researchers often need to develop custom devices, which not only increases the time required to initiate experiments but also compromises reproducibility across studies (NETRI, 2025; B. Zhang et al., 2018).

On the industrial front, the absence of dedicated platforms compounds the difficulties faced by researchers. While commercially available microfluidic devices have contributed significantly to other fields, no specialized devices currently exist for the integration of NRs and cardiac cells. Although some devices can be repurposed to study these interactions, the lack of tailored tools undermines the reproducibility, efficiency, and precision required for neuro-cardiac studies. The development of a specific platform designed for this purpose could open new avenues for targeted investigations and accelerate the pace of discovery in the field.

The development of neuro-cardiac OOC systems necessitates interdisciplinary collaboration that integrates expertise from neuroscience, cardiology, and bioengineering. Such integration requires not only specialized knowledge but also well-established collaborative infrastructures, which are often lacking in academic environments (Ardell and Armour, 2016). In addition, the differentiation and maturation of human-induced pluripotent stem cells into relevant neuronal and cardiac cell types remain costly both due to the high expense of reagents and culture media, and because of the need for highly trained personnel. Another significant challenge lies in the limited availability of validated biomarkers or assays for evaluating neuro-cardiac function in vitro. The absence of robust readouts hampers the ability to assess model fidelity and functional relevance, ultimately limiting their utility in translational research (Ottaviani et al., 2022).

Recent contributions to the field have stagnated, with the latest paper in this niche published in 2022. The limited availability of dedicated microfluidic platforms designed to model the neuro-cardiac junction may result from a confluence of technical, biological, and socio-scientific challenges. Neuro-cardiac communication involves complex bidirectional signalling through neurotransmitters (e.g., norepinephrine), ion channels, gap junctions, and electrical coupling. Accurately modelling this interface requires the co-culture of NRs and CMs that retain long-term functionality is particularly challenging when using human-derived cells such as hiPSC, which frequently display immature or inconsistent phenotypes (Burridge et al., 2014; Karbassi et al., 2020). Moreover, most existing OOC systems are tailored toward single organs or well-established organ pairings (e.g., gut–liver), whereas the NCJ is a relatively niche and less-characterized system, complicating the development of standardized readouts or stimulation protocols (B. Zhang et al., 2018). Another contributing factor lies in research funding trends, which have historically prioritized disease models with clear clinical translation pathways, such as cancer and diabetes. In cardiovascular research, attention has largely been directed toward heart failure and arrhythmias, often without incorporating autonomic regulation (Emulate Inc., 2025). As with all biological modelling approaches, ethical considerations also play a role in the field. Access to primary human NRs is severely restricted, and although hiPSC-derived NRs offer promise, their differentiation into mature, functionally integrated circuits remains inefficient, technically demanding, and costly. The insufficient research in the field presents a significant barrier to progress, limiting our ability to address critical gaps in foundational knowledge and therapeutic advancements. Challenges such as accurately replicating the complex interplay between the nervous and cardiac systems in vitro remain unresolved. This limitation hampers progress in understanding conditions like arrhythmias influenced by autonomic dysfunctions, where these systems interact intricately.

Microfluidic neuro-cardiac OOC rely on specialized cell seeding techniques such as manual loading via glass pipettes, which can significantly affect the accessibility and usability. These highly specific procedures often require trained personnel and specialized equipment, thereby raising the technical barrier to entry for researchers outside expert laboratories (Soucy et al., 2020; Virlogeux et al., 2018). Inconsistent seeding methods further contribute to variability in cell distribution and density across experiments, undermining reproducibility between studies and laboratories (Moutaux et al., 2018; Wevers et al., 2016). While automated and standardized seeding solutions have the potential to address these issues, they remain underutilized in current neuro-cardiac OOC systems, limiting scalability and ease of implementation (Axosim, 2025; Virlogeux et al., 2018).

It is likely that future platform designs should emphasize user-friendly interfaces, prefabricated seeding ports, and compatibility with standard laboratory equipment in order to improve the accessibility and adoption. Such features would reduce reliance on expert handling and enhance overall usability for a broader scientific audience. Additionally, fostering industry partnerships and promoting open-source initiatives could accelerate the development and dissemination of standardized tools, enabling wider use beyond specialized research environments.

Another critical limitation is the reliance on two-dimensional (2D) models in existing studies, which fail to capture the complexity of NCJs that function in a three-dimensional (3D) *in vivo* environment. While 2D systems have provided valuable initial insights, they are inherently limited in their ability to replicate the intricate structural and functional interactions of neuro-cardiac systems. Not only do 2D models suffer from a lack of spatial organization, but they do not also replicate the mechanical cues CMs and synapses exhibit *in vivo*. In physiological conditions, sympathetic and parasympathetic nerve terminals exhibit synapse-like formations with CMs in a spatially organized or oriented manner, which typifies physiological organization and is absent from conventional 2D cultures (Dokshokova et al., 2022; Kimura et al., 2012). In vivo, CMs respond to mechanical stimuli (e.g., substrate stiffness, topography, and cyclic mechanical strain). These contextualized cues largely lack in 2D systems, which can cause altered patterns of cell alignment, loss of contractility, and different gene expression profiles (He et al., 2020; L. Xu et al., 2021). NRs, for example, often have neurite outgrowth in a random fashion instead of neuro-organized synaptic networks in traditional 2D cultures which does not lend them for studies of synaptic modulation, neurotransmitter release kinetics, or mechanisms of long-term potentiation or depression (Franzoso et al., 2016). Less obvious than structural and morphological deficits in 2D models are inevitable deficits in electrophysiological dimensions. Functional parameters related to biology such as action potential propagation and intrinsic pacemaker activity have poor translation, limiting their use as predictive models for drug screening and disease modelling (Spijkers et al., 2021). As such, 2D platforms are not only inadequate representations of the three-dimensional *in vivo* neuro-cardiac junction, but they also lack the fidelity required to model key physiological and pathological processes.

Key cellular and molecular behaviours, as well as physiological events, are often overlooked in these oversimplified models. Moving towards 3D systems offers the potential to better approximate *in vivo* conditions, thereby enhancing the translational value of research findings.

The development of three-dimensional models, however, presents its own set of challenges, including the requirement for greater technical expertise and limitations in scalability. Despite these hurdles, such challenges can be mitigated through collaborative efforts between academia and industry. Encouraging partnerships and providing targeted funding support for innovative research could facilitate the design of advanced technologies specifically tailored for neuro-cardiac junction (NCJ) studies. These collaborations hold significant potential to accelerate the development of novel tools and methodologies that could revolutionize the field. Nevertheless, transitioning from 2D to 3D systems introduces additional technical barriers, such as limited optical access, increased variability in fabrication and culture conditions, and higher overall costs, which must be systematically addressed before 3D platforms can achieve widespread adoption.

Although 3D models offer enhanced physiological relevance, they also introduce significant technical and practical challenges. Constructing 3D environments that simultaneously support neuronal alignment and cardiomyocyte contractility requires the use of advanced biomaterials and fabrication techniques. Hydrogels, scaffolds, and bioprinting approaches must be carefully engineered to provide mechanical support while permitting neurite outgrowth and synchronized cardiac contractions (F. Zhang et al., 2021). The increased complexity of 3D systems also imposes constraints on imaging and data analysis. Advanced imaging technologies such as confocal microscopy or light-sheet fluorescence are often required to capture spatial dynamics within these structure technologies that remain inaccessible in many laboratories (Hajal et al., 2022; Phouphetlinthong et al., 2023). Additionally, 3D models tend to be more expensive and labour-intensive compared to their 2D counterparts. Customized moulds, perfusion systems, and controlled oxygen environments further add to the complexity, making high-throughput screening particularly challenging (Hetzel et al., 2023). Moreover, scalability is compromised by variability in materials, protocols, and equipment availability across different research settings, hindering consistent replication in both academic and industrial contexts (Virlogeux et al., 2018).

While many researchers recognize the potential of 3D models, there is a growing consensus that widespread adoption should be preceded by solutions to issues of reproducibility, cost-effectiveness, and standardization. As a result, there is increasing interest in hybrid strategies that combine the throughput advantages of 2D platforms with the biological fidelity of 3D systems, offering a pragmatic balance between innovation and practicality.

Neuro-cardiac OOCs, like many other types of OOCs, are faced with one major problem. Although electrophysiology assays are able to be performed easily with the implementation of MEA in the OOC platforms, most biochemical assays are not viable, as both the cell number and medium volume are low. Extraction of DNA or RNA would lead to very small samples, and the same could be said for protein or even for extracellular vesicles. To combat this issue, either the amount of OOCs would need to be increased, or potentially the size of the OOC, allowing for bigger seeding chambers that could hold more cells and bigger reservoirs, that could hold more medium.

Despite the current limitations (Table 5), the potential for impactful discoveries in neuro-cardiac research remains immense.

**Table 5:** Problems and limitations of microfluidic platforms for NCJ modelling.

Challenges and limitations	Limitations
Microfluidic platforms for NCJ modelling	Co-culture challenges with hiPSC-derived cells
	Lack of off-the-shelf platforms
	No commercial tools for NCJ modelling
Biological challenges	Immature phenotypes of hiPSC-derived cells
	Low number of validated biomarkers
	Niche nature of NCJs
Funding and ethics	Limited funding for NCJ research
	Ethical and logistical issues of hiPSC and Primary cell culture
Technical limitations	Lack of standardised seeding techniques
	Variability in cell distribution
	Lack of automated solutions
	Challenges in adhesion of immature neurons and cardiomyocytes
	Exploration of alternative seeding techniques for neurons and cardiomyocytes
Inadequacy of 2D models	Inadequate representation of 3D *in vivo* environments
	Limited predictive power
3D model problems	Higher technical expertise required
	Imaging and analysis constraints
	Higher costs and labour intensity
	Scalability issues
Biochemical assays	Limited sample volume in OOCs
	Solutions not yet scalable

Although microfluidic systems face several limitations, some inherent to the platforms and others due to model selection, they also offer numerous advantages that can be further optimized with ongoing technological advancements. A key benefit lies in their capacity to recreate dynamic microenvironments that closely resemble *in vivo* conditions. By incorporating fluid flow, shear stress, and concentration gradients akin to those found in the human body, most organ-on-a-chip systems enhance cell functionality and elicit more physiologically relevant biological responses compared to static 2D cultures or animal models (B. Zhang et al., 2018). Another major advantage is the use of human-based biology, particularly through the integration of human-induced pluripotent stem cells. While hiPSC-derived cells often exhibit immature phenotypes, this approach significantly reduces species-specific variability and improves translatability of experimental findings to human physiology, making OOCs highly valuable for drug testing and disease modelling applications compared to conventional animal models (Burridge et al., 2014; Karbassi et al., 2020).

The co-culture capabilities of OOCs further expand their utility by enabling the simultaneous cultivation of multiple cell types, such as NRs and CMs in precisely defined spatial arrangements. This allows for the investigation of complex intercellular interactions, particularly at the neuro-cardiac junction, where bidirectional signalling via neurotransmitters, ion channels, and electrical coupling plays a crucial role in cardiac regulation (Ardell and Armour, 2016; Zucker et al., 2012). Such co-cultures are essential for studying both the physiological and pathological dynamics of neuro-cardiac communication. Moreover, OOCs support the formation of 3D tissue architecture, offering a more accurate representation of native tissues than traditional 2D models. These three-dimensional structures facilitate appropriate cell alignment, extracellular matrix integration, and functional organization, key features necessary for recapitulating the mechanical and structural complexity of organs such as the heart and nervous system (Dokshokova et al., 2022; Kimura et al., 2012).

While the initial setup costs for OOC systems may be higher than those for conventional models, they offer potential long-term cost-effectiveness by reducing reliance on animal testing and enabling earlier identification of viable drug candidates. This can accelerate the drug discovery pipeline and ultimately reduce overall development costs (Hetzel et al., 2023).

Addressing the gaps in literature and developing specialized devices could unlock a deeper understanding of how the nervous system regulates cardiac function. These advancements would not only benefit basic research but also contribute to improved clinical outcomes for patients with complex neuro-cardiac conditions. A concerted effort to prioritize this field through innovation, collaboration, and resource allocation will be essential for realizing its full potential.

## 4 Future perspectives for microfluidics neuro-cardio models

Future prospects for the development of OOC in general, and cardiac OOC in particular, will focus on the three main axes of vascularization, innervation and instrumentation.

The vascularization of organoids and OOC is necessary to deeply irrigate tissues and cells. Spheroids tend to have necrotic tissues in the deepest layers due to a lack of diffusion of nutrients, but also of gases, necessary for cell development. Developing effective vascular networks in OOC is also essential to mimic the natural supply of oxygen and nutrients, which supports cell viability and function. Future efforts will focus on integrating hierarchical vascular networks using 3D bioprinting and endothelial cell co-culture to mimic native perfusion. The microfluidic patterning could enable controlled vessel formation, endothelial network and provide intravascular perfusion to the 3D structures with physiological blood flow velocities (Quintard et al., 2024). However, the engineered vessels may lack the hierarchical complexity (*e.g.,* arterioles, capillaries) and dynamic responses (*e.g.,* angiogenesis, shear stress adaptation) of native vasculature. Long-term stability and perfusion efficiency might still be constrained. Additionally, coupling vascularized neuro-cardiac models with blood-brain barrier (BBB)-on-chip systems (Hajal et al., 2022) may facilitate studies on neuroactive drug delivery and systemic crosstalk. The model integrates key BBB components (*e.g.*, endothelial cells, astrocytes, pericytes) in a microfluidic chip, mimicking the tight junctions, efflux transporters, and cellular crosstalk critical for BBB function. This improves drug permeability predictions compared to static 2D cultures. However, the model may lack immune cells (*e.g.,* microglia) and dynamic interactions with the neurovascular unit (*e.g.,* NRs), which regulate BBB function *in vivo*.

The innervation of organoids is the second line of research, where the innervation of muscle cells by motor neuron axons enables the latter to be electrically stimulated, and could help these cells to mature correctly. Motor control of CMs through the stimulated evocation of action potentials would enable control of the beating rate to be implemented in cardiac OOC. Finally, the instrumentation of cardiac OOC, which involves integrating the measurement of the excitation-contraction coupling including extracellular field potentials and mechanical contractions, is the last area of development. This involves the development of measurement structures such as microelectrodes for measuring local potentials, as well as mechanical structures such as strain gage beams or systems for optically reading cell cluster deformations to analyze the contractile functions of cardiac OOC.

Current models often simplify neural-cardiac interactions. Future platforms should incorporate heterogeneous neuronal populations (*e.g.,* sympathetic, parasympathetic, sensory NRs) to recapitulate autonomic regulation. Integrating neurotrophic factors and extracellular matrix gradients could further guide axon guidance and transport kinetic, improve secretory vesicles and mitochondrial transport and synaptogenesis (Moutaux et al., 2018).

Next-generation OOCs will integrate flexible electronics and AI-driven analytics for real-time, multimodal monitoring. Stretchable sensors and optical reporters could track contractility, electrophysiology, and neurotransmitter release simultaneously (Lind et al., 2017). Machine learning algorithms may decode complex datasets to predict drug responses or disease progression. Combining these tools with high-throughput microfluidic chips, such as the OrganoPlate, will accelerate preclinical screening (Wevers et al., 2016).

For a clinical point of view, patient-derived neuro-cardiac OOC systems are poised to make a profound impact on clinical research and patient care. By providing more accurate disease models, enabling personalized medicine, reducing reliance on animal testing, and facilitating the development of innovative therapies, these systems represent a significant advancement in biomedical science in agreement with the 3Rs. Their continued development and integration into clinical workflows will enhance the ability to diagnose, treat, and prevent cardiovascular and neuro-cardiac diseases, ultimately improving patient outcomes. They should facilitate the development of novel and more accurate therapies, including gene and cell-based treatments, by providing a controlled environment to test their effects on neuro-cardiac interactions. Neuro-cardiac OOC systems should assess the cardiotoxic effects of new drugs, helping to identify potential adverse effects early in the development process. In particular, they should allow for the evaluation of neurotoxic and cardotoxic effects, ensuring that new treatments do not adversely affect the nervous system and heart. Neuro-cardiac OOC may be used in the future to train medical students and professionals, providing a hands-on understanding of neuro-cardiac physiology and pathology. They may even offer a platform to simulate clinical scenarios, aiding in the development of clinical skills and decision-making.

## CRediT authorship contribution statement

**Maria João Ferreira:** Conceptualization, Investigation, Writing – original draft, Writing – review & editing. **Sarah Colombani:** Investigation, Formal analysis, Visualization, Writing – review & editing. **Albin Bernardin:** Methodology, Software, Validation, Data curation. **Alain Lacampagne:** Supervision, Project administration, Funding acquisition. **Jean-Luc Pasquié:** Resources, Data curation, Writing – review & editing. **Pedro F. Costa:** Conceptualization, Resources, Project administration. **Benoit Charlot:** Methodology, Validation, Supervision. **Albano C. Meli:** Conceptualization, Supervision, Writing – original draft, Writing – review & editing, Funding acquisition, All authors approved the final version of the manuscript.

## Declaration of competing interest

The authors declare that they have no known competing financial interests or personal relationships that could have appeared to influence the work reported in this paper.

## Data Availability

No data was used for the research described in the article.
